# Secretory breast carcinoma in a female adult with liver metastsis: a case report and literature review

**DOI:** 10.1186/s13000-021-01156-6

**Published:** 2021-10-10

**Authors:** Jing Lian, Li-Xia Wang, Jiang-hong Guo, Peng Bu, Yan-feng Xi, Ke-ming Yun

**Affiliations:** 1 Institute of Forensic Medicine, Shanxi Medical University, Yingze District, Shanxi Province Taiyuan, People’s Republic of China; 2 Department of Pathology, Shanxi Provincial Cancer Hospital, Xinghua ling District, Shanxi Province Taiyuan, People’s Republic of China

**Keywords:** Breast, Secretory breast carcinoma, Metastasis, ETV6-NTRK3, Case report

## Abstract

**Background:**

Secretory breast carcinoma is an uncommon subset of breast cancer that usually has a favorable outcome. Although initially described in children, it also occurs in adults where it may metastasize, possibly resulting in death. To date, only 20 cases of secretory breast carcinoma with distant metastases have been described.

**Case presentation:**

A 42-year-old female presented with liver metastasis after modified radical mastectomy of the left breast in 2008 at 34 years of age. The liver metastasis was morphologically similar to the primary tumor. Pan-TRK and Fluorescence in situ hybridization showed a rearrangement in the ETV6 gene. She subsequently underwent adjuvant chemotherapy with a fatal outcome.

**Conclusions:**

Although secretory breast carcinoma is usually associated with favorable outcomes, our study and reviews provide a novel insight into the genetic spectrum and treatment of secretory breast carcinoma showing reduced expression of hormone receptors, abnormal genomic profiles, and possible poor prognosis. Targeted therapy may curb clinically aggressive cases. Additional molecular investigations are needed to determine the links between specific mutations and poor prognosis.

## Introduction

Secretory breast carcinoma (SBC) is a rare form of breast cancer first described in 1966 as “juvenile breast carcinoma” in seven patients between three and fifteen years of age [1]. Subsequently, more adult cases were reported, and the distinctive histopathology of the tumor resulted in a change of name to “secretory carcinoma”.

SBC is composed of polygonal tumor cells characterized by intra- and extracellular secretions and cytoplasmic eosinophilic granules. Nuclear pleomorphism is almost always mild or moderate, and mitotic activity is low. There are four patterns that may exist in different combinations, namely, microcystic, solid, tubular, and papillary. Immunohistochemically, the tumor cells are generally estrogen receptor (ER) and progesterone receptor (PR) negative, and do not show amplification or overexpression of human epidermal growth factor receptor 2 (HER2) [2]. The tumor cells, however, strongly express S-100 and CK5/6 in a characteristic pattern [3]. The presence of a balanced chromosomal translocation, t(12;15)(P13;q25), resulting in an Ets variant 6-neurotrophic receptor tyrosine kinase 3 (ETV6-NTRK3) fusion gene was reported by Tognon et al. in 2002 [4]. It was further shown that SBC characterized by this fusion gene phenotypically resembles basal-like breast carcinomas, although the specific genotypic features of SBC differ from these cancers [5].

In spite of these abnormalities, SBC is usually an indolent tumor with a good prognosis, even in the presence of axillary lymph node involvement. Given the indolent behavior, the tumor is usually managed conservatively, primarily by surgical resection, and there are no specific guidelines for radiotherapy and chemotherapy, with varying reports in the literature.

Metastasis of SBC beyond the ipsilateral axillary lymph nodes is unusual. Only 20 cases of SBC with distant metastases have been described over the last 50 years [1, 3, 6–14]. Here, we report a case of a 42-year-old woman with SBC and liver metastasis after mastectomy and sentinel lymph node biopsy. We also review the literature and describe the clinical, pathological, and molecular attributes of this rare form of breast cancer.

## Case presentation

A 42-year-old Chinese lady had presented with a slowly enlarging left breast lump in 2008 at 34 years of age. Her ultrasound showed a 4.69 cm×2.85 cm solid, irregular mass in the outer upper quadrant of the left breast. She had no history of breast trauma, discharge, nipple inversion, pain, or other symptoms. She also had no family or past history of cancer. She was admitted to Shanxi Provincial Cancer Hospital. A lumpectomy in August 2008 resulted in an initial intraoperative diagnosis of invasive carcinoma. Subsequently, she underwent a modified radical operation with axillary lymph node dissection. Her diagnosis was invasive ductal carcinoma, grade II, and axillary lymph node-negative. She received two chemotherapy cycles of 5-fluorouracil, pirarubicin, and cyclophosphamide, four cycles of doxorubicin and cyclophosphamide, and radiation therapy. The patient’s condition was stable after treatment. In 2016, the abdominal CT scan showed multiple nodular low-density shadows in the liver, with a maximum cross-section of 8.2 × 4.1 cm.

**Fig. 1:**
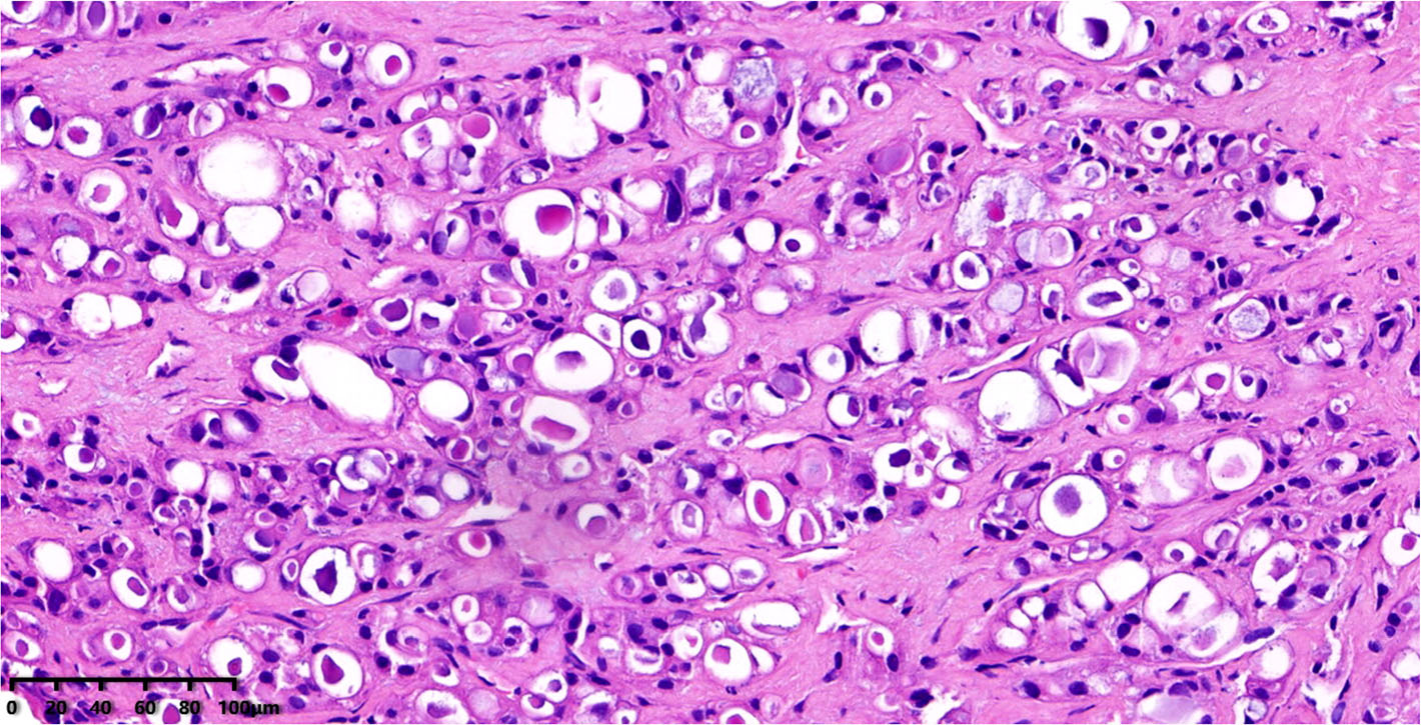
The metastatic carcinoma in liver. HE 200×

**Fig. 2:**
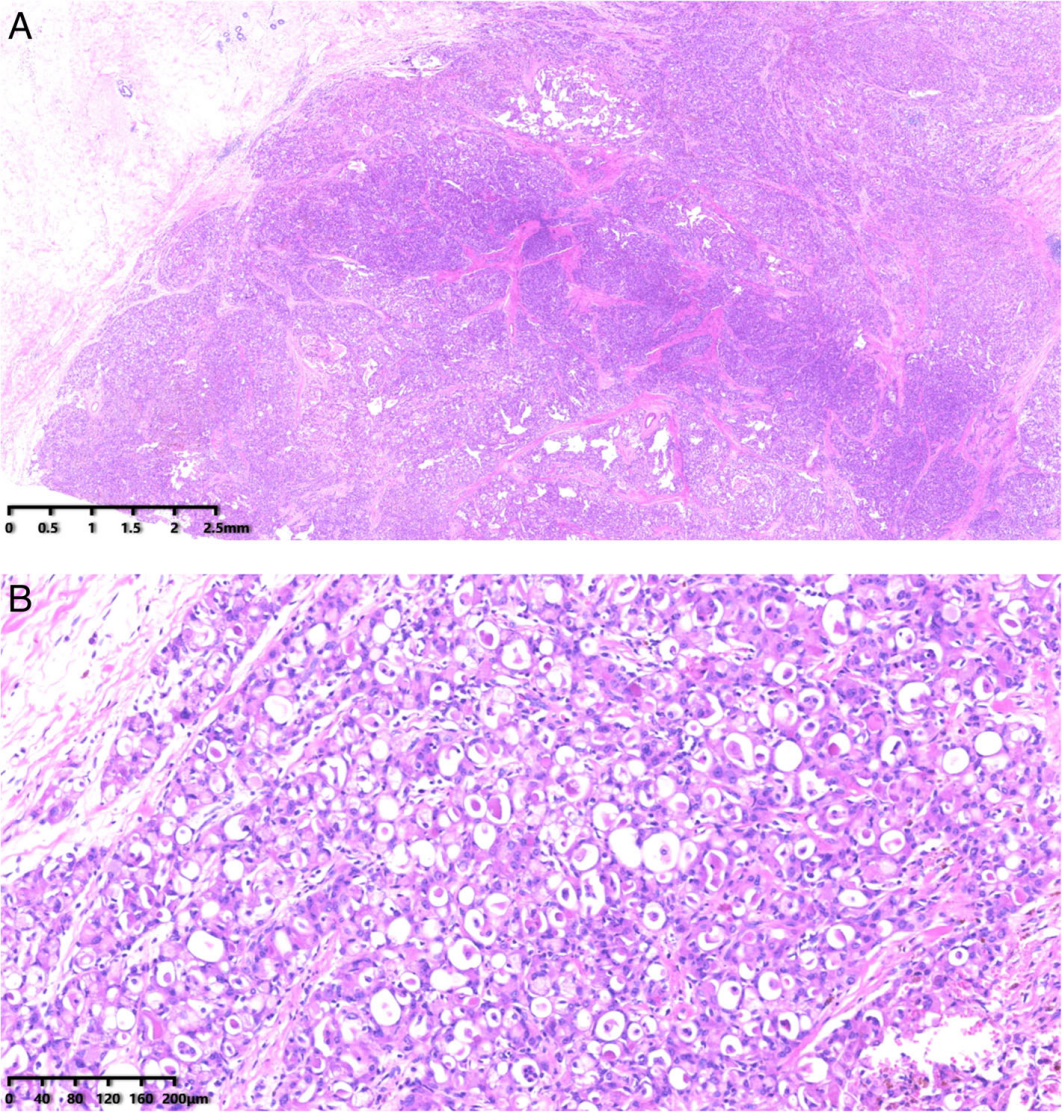
**A** The primary tumor in breast. HE 10×. **B** The primary tumor in breast. HE 100×

Microscopically, the tumor cells were arranged in tubular, solid, and microcystic patterns. There was widespread intracellular and extracellular secretory material. The cell morphology was mild-to-moderately atypical with the presence of eosinophilic granules and foamy cytoplasm. The nuclei were small and oval to round in shape with prominent nucleoli in some cases. There was little evidence of mitosis. The morphology of the metastatic carcinoma in the liver (Fig. 1) resembled that of the primary tumor in the breast (Fig. 2).

**Fig. 3:**
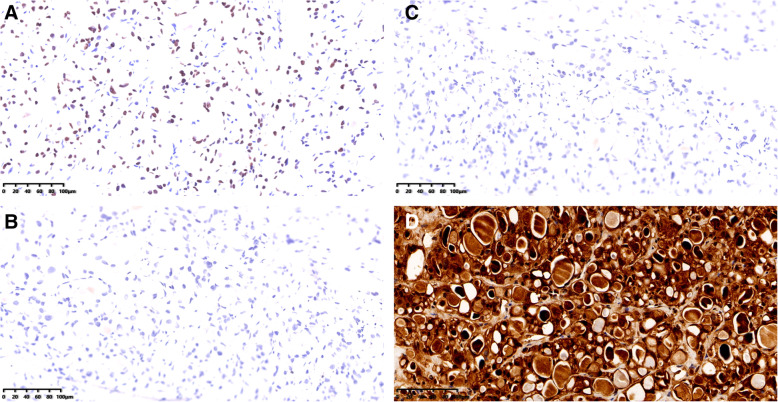
**A** Tumor cells were positive for GATA3. IHC 200×. **B** Tumor cells were negative for ER. IHC 200×. **C** Tumor cells were negative for ER. IHC 200×. **D** Tumor cells were positive for S-100. IHC 200×

**Fig. 4:**
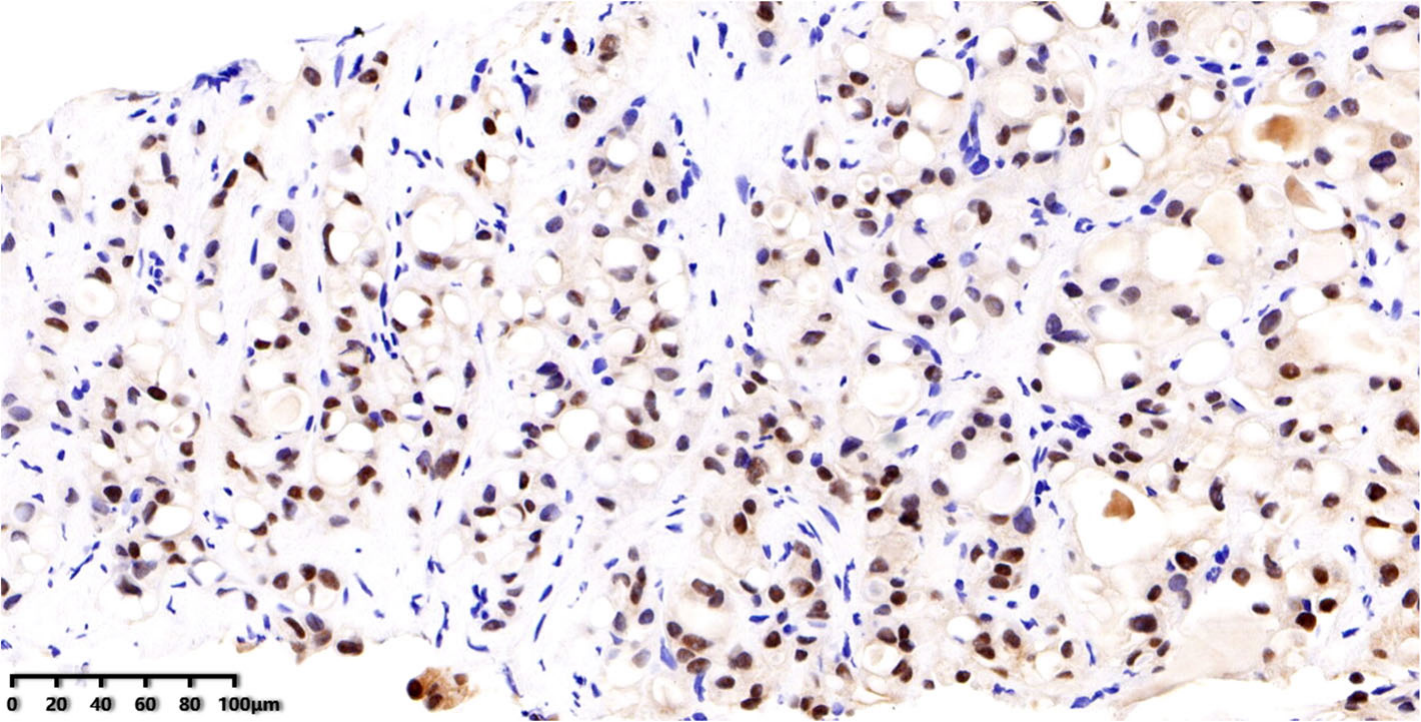
Tumor cells were positive for pan-TRK. IHC 200×

The panel of immune-histochemical stains included ER, PR, HER2, CK5/6, S-100, Ki67, GATA3(Fig. 3A), and pan-TRK. The tumor cells were basal-like in appearance and were negative for ER(Fig. 3B), PR(Fig. 3C), and HER2 and positive for CK5/6. The cells were also positive S-100 protein(Fig. 3D), the KI67 index was 10 %, and were positive for pan-TRK (Fig. 4), suggesting an ETV6 gene rearrangement.

**Fig. 5:**
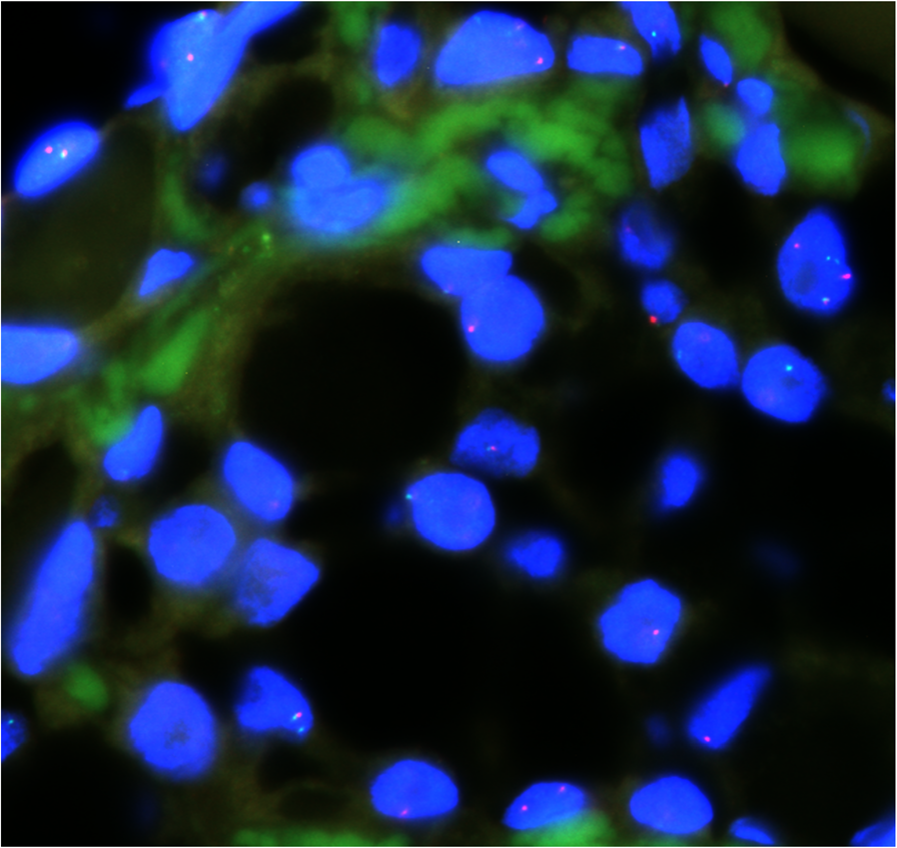
Tumor cells were shown to have rearrangement of the ETV6 gene. FISH 1000×

Mutational analysis using a dual-color break-apart probe showed an ETV6 gene rearrangement (Fig. 5) characterized by more numerous ETV6-specific split signals above the cut-off value (10 %).

A diagnosis of SBC was made on the basis of these findings.

The patient was followed up until her death 108 months later. Data were collected telephonically and these, together with the data of the initial diagnosis, were used for calculation.

## Discussion

SBC is a malignant breast tumor initially described in children. It is extremely rare and accounts for less than 0.02 % of all breast cancers [15]. It usually presents early and has an indolent clinical course and excellent prognosis. In appearance, the tumor is first seen as a small, apparently benign nodule with microcalcifications and spiculated margins visible on ultrasound [3, 16]. As these features may apply to benign lesions as well as some other well-circumscribed breast carcinomas, it is often difficult to distinguish SBC from other breast cancers. However, the microscopic appearance of secretory material, both intra- and extracellular, is characteristic of SBC. The cell nuclei were small, oval, or round, and a prominent nucleolus may be visible. There was little or no evidence of mitosis, necrosis, or vascular invasion. An additional characteristic of SBC is its association with in situ carcinoma, in contrast to other basal-like cancers [5, 17].

Earlier studies have found that SBC patients are almost exclusively negative for hormone receptors [18, 19], including the present case. However, two larger studies observed ER and PR positivity [2, 3] suggesting that there is more variation than originally thought. The HER2 status was, however, similar in all studies [2, 3, 18, 19]. All cases showed positive staining for S-100 protein and a low proliferation index, without increased Ki67 expression [2, 18, 19]. The t(12;15) translocation, producing an ETV-NTRK3 fusion that encodes a chimeric tyrosine kinase and is characteristic of SBC, has also been recorded in the pediatric mesenchymal tumors cellular mesoblastic nephroma and infantile fibrosarcoma [5, 20] where the chimeric kinase appears to be responsible [14]. It has been found that more than 90 % of SBC cases showed this translocation [4, 11]. This characteristic expression of the ETV6-NTRK3 fusion gene may be useful as a biomarker for the differential diagnosis of SBC from other breast carcinomas. In this respect, Pan-TRK IHC is a new sensitive and specific marker for SBC that recognizes a conserved sequence of TRK and can provide a more rapid and cost-effective test than ETV6 FISH, as an adjunct in the diagnosis of SBC. In terms of histology and immunohistochemistry, the presence of eosinophilic granules and foamy cytoplasm and ER, PR, and HER2 negativity with S-100 positivity, respectively, assist with the diagnosis. ETV6 FISH can also be used to distinguish SBC from other disorders, including benign conditions such as juvenile papillomatosis with apocrine metaplasia or mucinous carcinoma, and microglandular adenosis or malignant lesions such as acinic cell carcinoma, cystic hypersecretory carcinoma, and apocrine carcinoma that have similar morphology and are ER, PR, and HER2-negative.

The metastatic lesions and axillary lymph nodes in SBC show comparable secretory and growth behavior to the primary tumor. Lymph node metastases have been found in approximately 30 % of cases [19]. Although metastasis beyond the lymph nodes is extremely rare, it is generally fatal. Our literature review revealed 20 cases of SBC in PubMed. The relevant clinical and pathological information of these cases is summarized in Table 1. Eleven of the cases were women and four were men, with ages between 8 and 73 years with a median age of. 35.5 years (mean=26 years). While SBC affected women over a wide age range (8-73 years), male patients tended to be younger (20-52 years). Detailed clinical information was not available for another six patients [3]. The median size of the primary tumor size was 5.3 cm(mean=5.93 cm; range=2.0-12 cm). Surgery, adjuvant chemotherapy, and postoperative radiotherapy were used in the patients. At presentation, fourteen patients underwent mastectomy or breast-conserving surgery. In six of 10 cases, biopsy at the time of initial diagnosis identified lymph node involvement. Six patients received chemotherapy and three received radiotherapy. The median time between diagnosis and metastasis was 25 months (mean=55 months; range=2.5-240 months) with most metastases seen in the lung as well as in the liver and bone. The median duration of follow-up was 50 months (mean=71 months; range=6-240 months) and 14 patients died from metastatic disease.

**Table 1:** Review of clinical data of 21 cases of SBC with distant metastasis (including one case from our study)

Authors (publication year)	Gender(F/M)	Age (year)	Tumour size(cm)	Status of axillary lymph nodes	treatment	ETV6 rearrangement (FISH or/and RT-PCR)	Targeted therapy with pan-TRK (N/Y)	Interval diagnosis of primary and metastasis(month)	Site of metastasis	outcome	Time after initial diagnosis (month)
Tavassoli and Harris (1980) [1, 21]	F	25	6.0	8/14	RM	NE	N	not reported	disseminated	died of disease	10
Tokunaga, et al. (1985) [1, 22]	F	13	4.5	not sampled initially	SM	NE	N	72	kidney, lungs, mediastinum, pancreas, pleura	died of disease	108
Nguyen and Neifer (1987) [1, 23]	F	73	2.0	not sampled initially	BCS	NE	N	18	disseminated	died of disease	35
Kramusz, et al. (1989) [1, 24]	M	24	4.0	not sampled	SM+RT	NE	N	240	liver; skin	died of disease	240
Krohn, et al. (1989) [1, 25]	F	16	11.0	1/28	NAC+MRM+AC	NE	N	2.5	lung	not reported	not reported
Herz, et al. (2000) [6]	F	15	not reported	0/5	MRM	NE	N	144	lung	died of disease	174
Woto-Gaye, et al. (2004) [7]	M	20	12.0	6/6	MRM	NE	N	5	lung, liver	died of disease	6
Arce, et al. (2005) [1, 26]	M	52	7.0	2/24	MRM+AC+RT	present	N	18	lung	alive with disease	25
Anderson, et al. (2006) [1, 27]	F	61	8.0	+	NAC+MRM	NE	N	7	bone,lung	alive with disease	not reported
Wong, et al. (2012) [10]	F	68	6.0	0/16	SM+ALND+RT	present	N	10	lung	died of disease	13
Del Castillo, et al. (2015) [11]	F	62	4.1	2/12	MRM+ALND+RT+AC	present	N	7	bone, liver, lung, skin, serous	died of disease	11
Neerav Shukla, et al. (2017) [12]	F	8	not reported	not reported	BCS+AC	present	Y	72	lung, bone	alive with disease	84
Yosef Landman, et al. (2018) [1, 28]	F	36	not reported	not reported	BCS	preaent	Y	32	lung, bone	alive with disease	56
Raza S Hoda, et al. (2019) [1]	M	26	2.0	0/5	SM+SLNB	present	Y	45	bone	alive with disease	50
Lijuan Li, et al.(2019)^a^ [3]	not reported	not reported	not reported	not reported	not reported	present	N	not reported	Bone(3/6), liver(1/6), contralateral supraclavicular lymph node(2/6)	five patients died of disease(5/6)	not reported
Present study	F	34	4.6 × 2.8	Negative	RM+AC+RT	present	N	96	liver	died of disease	108

*AC *Adjuvant chemotherapy, *ALND* Axillary lymph node dissection, *BCS* breast-conserving surgery, *F* Female, *M* Male, *MRM* Modified radical mastectomy, *NAC* Neoadjuvant chemotherapy, *NE* Not examined, *RM* Radical mastectomy, *RT* Radiotherapy, *SM* Simple mastectomy, *SLNB* Sentinel lymph node biopsy

^a^Six SBC patients demonstrated evidence of distant metastasis, but detailed clinical information was not available

The size of a breast cancer tumor is recognized as one of the major prognostic factors. In SBC, tumor size represents aggressive growth [1]. Our review of the SBC literature, as well as our own case, indicated a median size of 5.3 cm for clinically aggressive tumors, larger than other breast cancers. This may be because it is often misdiagnosed as a benign lesion, such as fibroadenoma, on imaging as it has a well-circumscribed, hypoechoic mass, sometimes with microlobulations. However, further studies are needed to confirm the relationship between tumor size and underlying molecular mechanisms in aggressive SBC cases.

Due to both its slow growth and optimistic prognosis, there are no specific guidelines for treating SBC. In addition, although surgery is recommended as the primary treatment, there are no guidelines concerning the extent of surgical resection. Conservative resection, radical mastectomy, and modified radical mastectomy are usually performed in adults with simple mastectomy, local excision with sentinel lymph node biopsy, and complete axillary dissection in children. Furthermore, there is no consensus on the necessity and usefulness of adjuvant chemotherapy and radiotherapy.

Breast cancer is known to be extremely heterogeneous, likely due to differences in the underlying molecular mechanisms. Immunohistochemical techniques are commonly used for identifying molecular subtypes, such as HER2 overexpression, luminal A and B, and differentiating between basal-like and normal breast-like tumors, as gene chip techniques tend to be both costly and inconvenient. SBC is usually immunochemically positive for S-100 and negative for ER, PR, and HER2, while also expression markers, such as CK5/6, which are frequently associated with basal-like tumors. However, SBC differs from basal-like tumors that are typically characterized by high histological grade, lymphocyte infiltration, cellular pleomorphism, necrosis, and high levels of proliferation while SBC shows only mild to moderate dysplasia without necrosis or excessive proliferation. In addition, local SBC tumor recurrence and distant metastases are not apparent after relatively short follow-up periods. SBC is one of the basal-like carcinomas that have a good prognosis. However, our literature review of SBC found that 14 SBC patients (14/19, 74 %) died from metastatic disease, suggesting a poor long-term prognosis. Future studies are required to determine the prognostic consequences of the basal-like marker expression in SBC as well as conducting long-term clinical follow-ups.

Analyses showed an ETV6 rearrangement in 13 cases, of which three cases were treated with a human tropomyosin-related kinase (TRK) inhibitor and were all still alive. No other molecular changes have been reported for SBC with distant metastases. The TRKs are neurotrophin receptors, with the three TRK isoforms encoded by the NTRK genes. Rearrangements are relatively common in these genes and may confer oncogenic potential on their protein products. Thus, targeting the TRKs may have therapeutic value in cancers, such as SBC, with these rearrangements. Such treatment can be performed relatively early, as proposed by Landman, et al [14] rather than waiting to determine the efficacy of other treatments. Thus, clinicians should be aware of this treatment option and should test for TRK fusion.

## Conclusions

As SBC is rare, there is a paucity of knowledge on its pathology, clinical behavior, and outcomes. We searched for and identified all reports of metastatic SBC in the literature to identify the characteristics of aggressive cases. These findings offer insight into both the genetic background and clinical treatment of SBC. SBC patients should receive appropriate management from multidisciplinary treatment planning conferences in order to obtain a favorable prognosis. Therapeutic targeting of the cancer offers promise for treating aggressive SBC cases. Further investigation is needed to fully understand the genetics and molecular mechanisms involved in SBC, particularly mutations linked to aggressive tumor behavior.

## Abbreviations

SBC: Secretory breast carcinoma
ER: Estrogen receptorand
PR: Progesterone receptor
HER2: Human epidermal growth factor receptor 2
ETV6-NTRK3: ETS variant 6-neurotrophic receptor tyrosine kinase 3
